# From the Behavioral Pharmacology of Beta-Carbolines to Seizures, Anxiety, and Memory

**DOI:** 10.1100/tsw.2007.48

**Published:** 2007-02-19

**Authors:** Patrice Venault, Georges Chapouthier

**Affiliations:** Vulnérabilité, Adaptation et Psychopathologie, CNRS UMR 7593, Hôpital Pitié-Salpêtrière, 91 Bd de l'Hôpital, 75634 Paris cedex 13, France

**Keywords:** GABA-A, benzodiazepine receptor, beta-CCM, methyl beta-carboline-3-carboxylate, selected strains, mouse, behavior, neurogenetics, pharmacogenetics, epilepsy, seizures, anxiety, learning, memory, aggression, balance disorders

## Abstract

A number of beta-carbolines are inverse agonists of the GABA-A receptor complex, acting on the benzodiazepine site. They show convulsive properties when administered at high doses, anxiogenic properties at moderate doses, and learning-enhancing effects at low doses. These data suggest a possible physiological relationship, through the GABA-A receptor channel, between memory processes, anxiety, and ultimately, in pathological states, epileptic seizures. This relationship seems to be confirmed partially by experiments on mouse strains selected for their resistance (BR) and sensitivity (BS) to a single convulsive dose of a beta-carboline. These two strains also show differences in anxiety and learning abilities. However, some opposite results found while observing the behavior of the two strains suggest that in addition to pharmacologically induced anxiety, there is spontaneous anxiety, no doubt involving other brain mechanisms.

## INTRODUCTION

Beta-carbolines are molecules that act on the benzodiazepine site of the GABA-A complex receptor. Some of them, such as abecarnil[1,2] or harmaline[3], have a systematic or occasional agonist action, similar to the action of benzodiazepine agonists. Others, however, have a clear inverse agonist action, i.e., they possess properties opposite to the properties of benzodiazepines[4,5,6]. These inverse agonist beta-carbolines are, therefore, powerful tools for analyzing the involvement of the GABA-A receptor complex in several behavioral situations. Of the different inverse agonist beta-carbolines, e.g., propyl beta-carboline-3-carboxylate (beta-CCP), methylamide beta-carboline (FG 7142), methyl-6,7-dimethoxy-4-ethyl-beta-carboline-3-carboxylate (DMCM), beta-carboline-3-carboxylate-*t*-butyl ester (beta-CCt), or 6-methoxy-1,2,3,4-tetrahydro-beta-carboline (pinoline), one is classically used for experimental research, i.e., methyl beta-carboline-3-carboxylate (beta-CCM).

The benzodiazepine site has three classes of ligand: (1) agonists, such as benzodiazepines; (2) inverse agonists, such as beta-CCM; and (3) "antagonists", such as Ro 15-1788 or flumazenil (Anexate®)[7]. Antagonists are assumed to have no effect when administered alone, but, on the site, can counter the effects of agonists[8] and inverse agonists[9]. However, this is only partially true, and a "perfect antagonist" with no effect of its own has yet to be found. Flumazenil, for example, while an excellent antagonist in most situations, still has effects of its own. In several learning situations[10,11,12,13,14,15], flumazenil can produce effects similar to the effects of an inverse agonist, but in some studies of the anticonvulsive action of benzodiazepine[16,17,18], flumazenil at very low doses has been seen to potentiate the anticonvulsive effect of diazepam, thus showing that it has a partial agonist action.

We will first describe key behavioral situations affected by an inverse agonist beta-carboline, comparing its action with the effects of agonists (such as benzodiazepines), antagonists (such as flumazenil), and of other convulsant agents acting on other sites of the GABA receptor complex (such as pentylenetetrazol). We will then show the relevance of two mouse strains selected for their sensitivity and resistance to beta-CCM when used to induce seizures. Our purpose here is not to provide a general review of the field, but rather to present an argument for a probable link between three essential brain functions: seizuring susceptibility, anxiety, and memory processing. For a general survey of the field, see the excellent reviews by Stephens et al.[19], Scott et al.[20], Maubach[21], and Rudolph and Möhler[22].

## BEHAVIORAL EFFECTS OF BETA-CCM AND RELATED COMPOUNDS

### Effects on Seizures

Benzodiazepines and GABA-A receptor agonists have a recognized anticonvulsive action and are routinely used for antiepileptic treatment[23,24,25,26], while inverse agonist beta-carbolines are convulsant or proconvulsant agents. Some beta-carbolines, such as DMCM, are powerful convulsants[27] whereas other compounds, called "proconvulsants", such as Ro 15-4513 (a partial inverse agonist), n-butyl beta carboline-3-carboxylate, and FG 7142, produce no spontaneous convulsive effect, but simply potentiate seizures induced by other compounds[28,29,30]. This seizuring effect has been studied extensively with beta-CCM, which is convulsive or proconvulsive in rats[31], mice[32], and chicks[33]. The effects can be countered by administering an agonist[31] or an antagonist, such as flumazenil[32]. In the course of a classical beta-carboline–induced seizure in mice, three stages of increasing intensity can be observed: first, myoclonic tremors of the limbs and vibrissae; then the full tonic-clonic convulsion, with the mouse falling on its side; and then, although rarely (if the dose administered is too high), a tonic convulsion with all four limbs pointing towards the rear and often leading to death. For obvious ethical reasons, the last stage should be avoided whenever possible. Electroencephalographic observations (see Fig. 1) show low-level spiking activity in the cortex and the hippocampus after administration of a low dose of beta-CCM (1 mg/kg, i.p.) and after the seizure triggered by a high dose (10 mg/kg, i.p.). During the behavioral seizure itself, spiking activity is very high (electroencephalographic seizure) in both the cortex and the hippocampus.

These responses have been observed after administering single doses of beta-CCM, but a number of studies have reported kindling effects. An early investigation of the beta-carboline FG 7142 by Little et al.[34] reported interesting kindling effects, and in both mice and rats[35,36,37,38], repeated administration of very low doses of beta-CCM, clearly insufficient to induce seizures as a single dose (1 mg/kg, i.p., in mice), has been shown to produce very powerful convulsions after several days, a typical kindling situation. Studying mice, Rössler et al. showed that there was gradual adaptation of brain GABA receptor binding before the convulsions appeared (a steady decrease of Bmax and a steady increase of Kd), suggesting "silent" biochemical preparation of beta-CCM kindling, even before behavioral symptoms (seizuring) occurred[35].

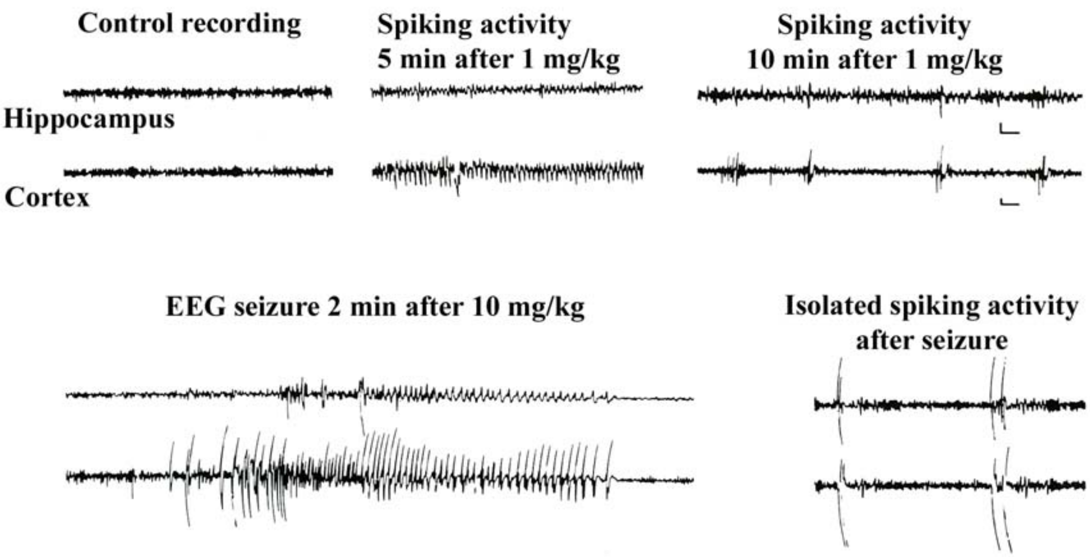

**FIGURE 1.** Beta-CCM–induced seizures: electroencephalographic data. With a dose of 1 mg/kg, i.p., no seizure occurred, but mild spiking activity could be observed in both the cortex and hippocampus. With a dose of 10 mg/kg, i.p., pronounced spiking activity was observed during the seizure, followed by occasional spikes during the postictal period. (Adapted from Prado de Carvalho et al.[32].)

## Effects on Anxiety

Benzodiazepines produce anxiolytic effects in both humans[39,40] and animals[41], while certain beta-carbolines produce anxiogenic effects in humans and animals. Studying humans, Dorow et al.[42] found evidence of an anxiogenic action of beta-carboline FG 7142 in healthy human volunteers. Animal studies have shown that certain beta-carbolines have an anxiogenic affect on monkeys[43] and rodents[10,39,40,41,42,47,48] (see Figs. 2 and 3 for examples.)

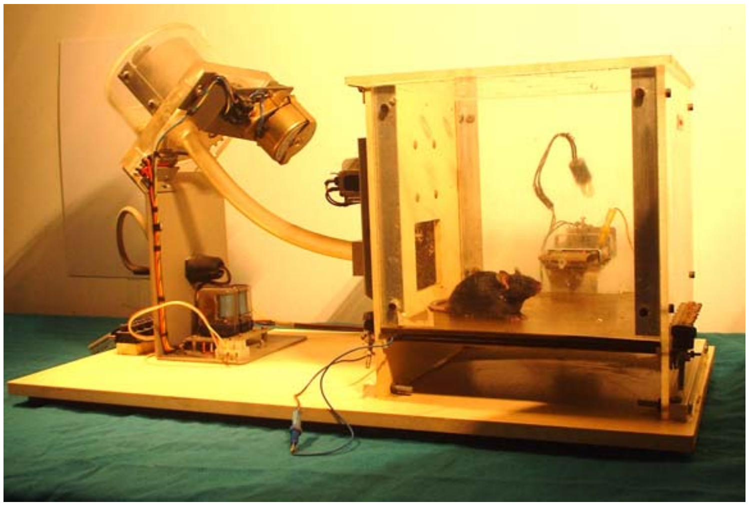

**FIGURE 2.** Mice in a conflict situation**.** In a Skinner box, a mouse can obtain a food reward by pressing a lever. In a conflict situation, when a light is on and when the mouse presses the lever, it obtains the food reward and also a mild electric shock via the metal floor and the lever. Anxious mice pressed the lever less frequently in the conflict situation, but there was little or no change to the lever pressing by nonanxious mice. (Copyright Venault and Chapouthier, CNRS.)

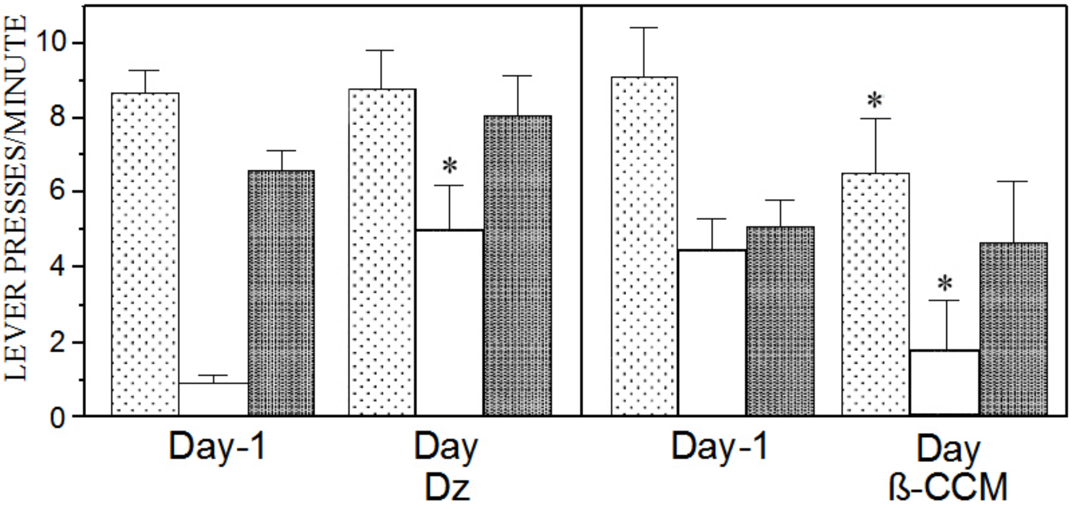

**FIGURE 3.** Treated mice (diazepam, beta-CCM) in a conflict situation**.** The middle column of each group of three is the conflict situation, i.e., with electric shock, while the first and third columns are the nonconflict, positively reinforced sessions, without electric shock. With a moderately high electric shock conflict situation, there was a sharp increase in lever pressing by mice after administration of diazepam (DZ), when compared to the previous day (left, middle white column), providing evidence of the anxiolytic effect of diazepam. With a milder electric shock conflict situation, there was a sharp drop in lever pressing after administration of beta-CCM (β-CCM), compared to the previous day without treatment, thus providing evidence of an anxiogenic effect of diazepam. For other significant effects, see Prado de Carvalho et al.[46]. (Adapted from Prado de Carvalho et al.[46].)

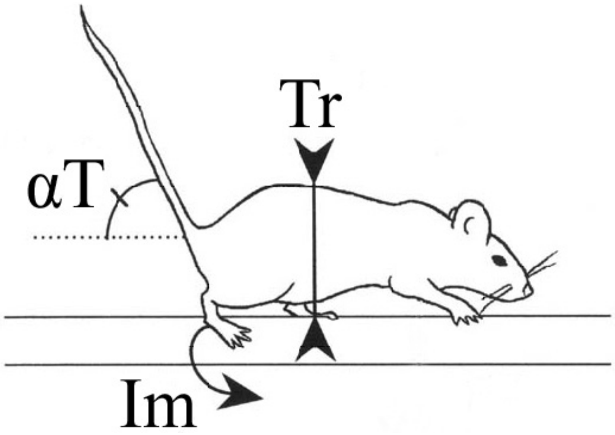

**FIGURE 4.** Assessment of performance on a rotating beam**.** Performance was assessed by the number of falls, the number of imbalances (Im), the angle of the tail (αT), and the elevation of the trunk (Tr). (Adapted from Lepicard et al.[53].)

For the purpose of comparison, it should be noted that convulsant compounds not acting on the benzodiazepine site, e.g., pentylenetetrazol and picrotoxin, have clear anxiogenic effects on monkeys[50] and mice[45,51] at doses lower than convulsive doses[52]. This showed that the efficient dose for all compounds producing anxiogenic effects is lower than the dose required for inducing convulsions.

The anxiogenic action of beta-CCM was assessed in an original task relating anxiety and balance control[53]. Mice were tested in an experiment that required them to walk on a long rotating rod to obtain a food reward. Anxious BALB/cBY mice fell frequently, while nonanxious C57Bl6/J mice walked along the rod without falling. Several behavioral parameters discriminate the two strains (see Fig. 4). It is interesting to note that the anxiolytic agent diazepam (0.5 mg/kg, i.p.) reduced the number of falls by

BALB/cBY mice and produced an overall improvement in behavioral parameters for anxious behavior, while beta-CCM (1.5 mg/kg, i.p.) produced more falls in C57Bl6/J mice and more anxious results (see Fig. 5A–D).

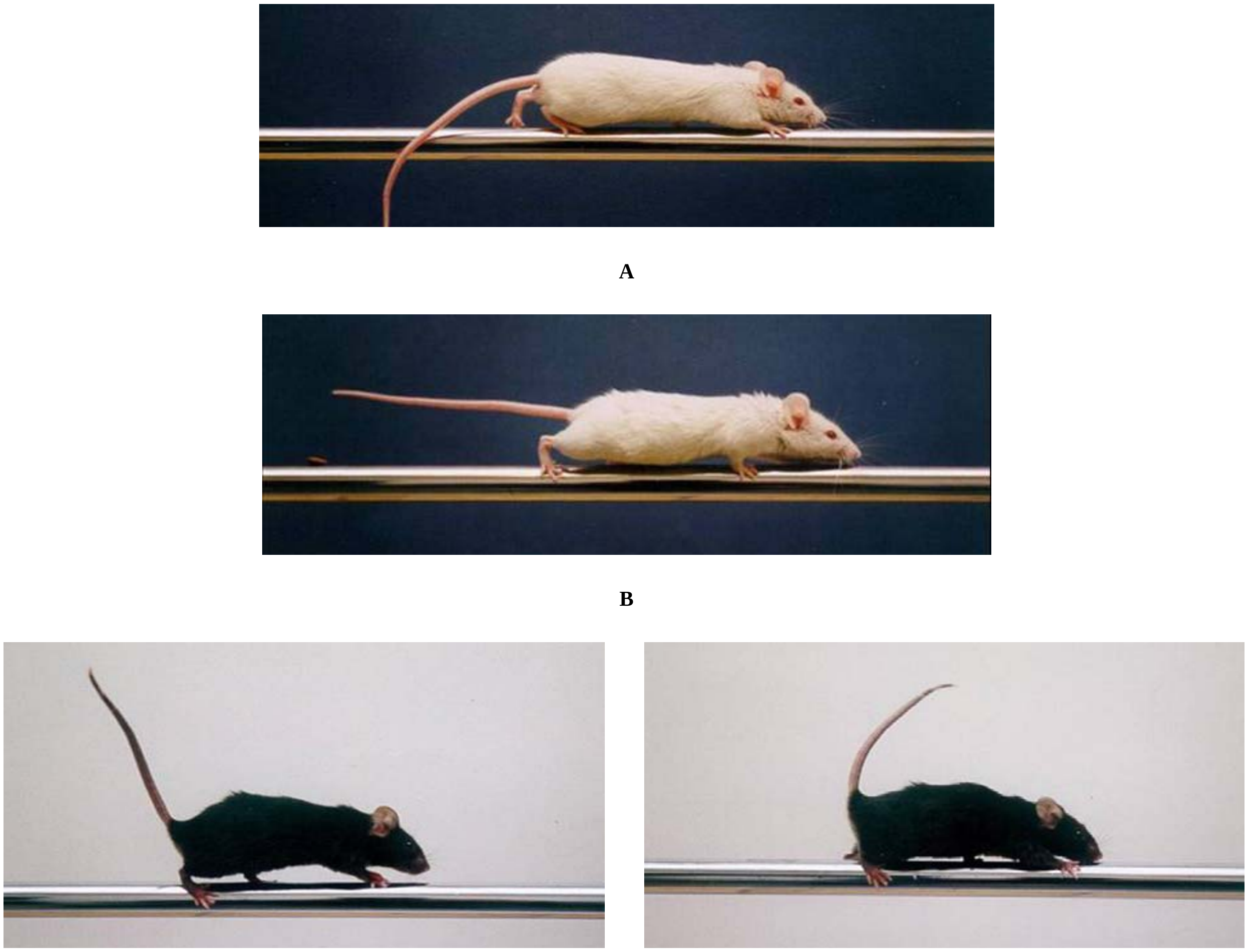

A

B

C D

**FIGURE 5.** Effects of diazepam and beta-CCM on rotating beam performance**.** (A) Anxious BALB/c mice recorded a higher number of imbalances (at least one paw losing contact with the bar) and more falls; the trunk was low and the angle of the tail was narrow. (B) All these parameters were reversed by the administration of the anxiolytic, diazepam (0.5 mg/kg, i.p.). (C) Nonanxious C57Bl/6J mice walked along the rotating rod without any imbalances or falls, using their tails to balance, keeping their trunks well above the rod, and keeping their tails rigid and at a high angle from the horizontal. (D) This was substantially reversed by the administration of an anxiogenic, beta-CCM (1.5 mg/kg, i.p.); the mice lost their balance, fell, kept low, and lost the rigidity of their tails, thereby losing the ability to use the tail to balance the body; they did not, however, reduce the tail angle significantly. (Adapted from Lepicard et al.[53].)

## Effects on Memory

Benzodiazepines are often said to have “amnesic” effects in humans. In the 1960s, anesthetists reported that benzodiazepines induced marked amnesia in patients, but only for the period after the drug was injected, i.e., anterograde amnesia[54,55]. Since then, several amnesic effects of benzodiazepines (treatment with diazepam, lorazepam, and triazolam) have been described in clinical practice[56,57] and in experimental studies on normal healthy volunteers[58,59,60]. Anterograde amnesia induced by benzodiazepines has also been observed in animals[61,62,63,64,65,66].

Benzodiazepines have “amnesic” effects, so the inverse agonists should then have inverse, i.e., “promnesic” effects. Several beta-carbolines, such as FG 7142, ZK 93426, and DMCM, have been shown to modify learning abilities in rodents[67,68,69,70,71]. Extensive studies of beta-CCM have shown that it improves learning in mice as assessed on several learning tasks: one-trial learning tasks, e.g., habituation to a new environment or passive avoidance[72,73], spontaneous alternation[10], and learning tasks over a series of trials[12]. An example of the promnesic action of beta-CCM is seen in Figs. 6 and 7. Doses needed to produce a promnesic effect are substantially lower than anxiogenic doses[52] (0.3 mg/kg instead of approximately 1 mg/kg for an anxiogenic effect). A similar promnesic action[72,75] has been observed after acute administration affecting imprinting in chicks, a phenomenon first described by Konrad Lorenz. Along the same line, “paradoxical” memory-enhancing effects of benzodiazepines have also been reported in some cases[74].

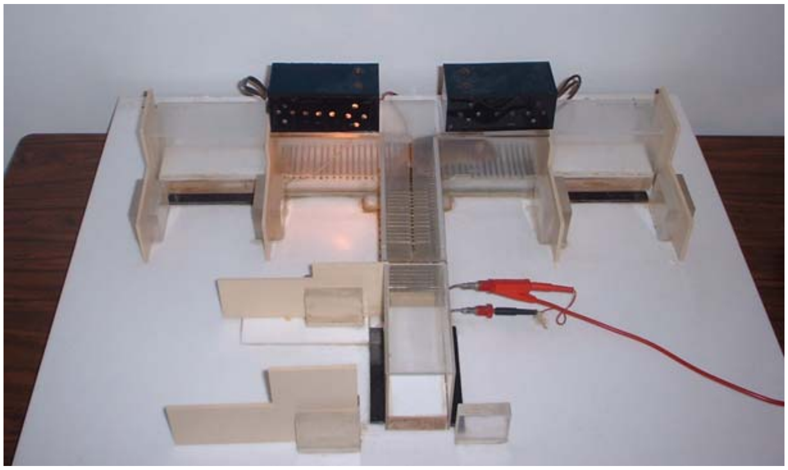

**FIGURE 6.** Light-dark choice situation in a T-maze**.** The target arms, left and right, can be lit according to a random sequence so that the mouse learns to choose the dark arms systematically. Complete learning required 60 trials over 6 consecutive days (10 trials per session). (Copyright Venault and Chapouthier, CNRS.)

Mayo et al.[11] pursued an hypothesis first made by Sarter et al.[76] and found that direct intracerebral administration of beta-CCM (at extremely low doses: 3 µg/0.5 µl) injected into the nucleus basalis magnocellularis of the rat enhanced recognition in a two-trial recognition task. This result is particularly interesting as the nucleus basalis magnocellularis of the rat is the equivalent of the nucleus basalis of Meynert in humans, which plays a clear role in mnesic impairments observed in Alzheimer-type senile dementia[77,78]. This identification of GABAergic neurons in the nucleus basalis magnocellularis being involved in memory processes offers new evidence for the putative role of GABA in Alzheimer’s disease.

As mentioned above, the antagonist flumazenil enhances learning in several situations: brightness discrimination in a T-maze with negative reinforcement (mice)[12], a brightness discrimination task with positive or negative reinforcement (mice)[13,14], a swim-escape task (rats)[15], step-down inhibitory avoidance and habituation to a buzzer test (rats)[79], and radial maze tests (rats)[80]. In these learning situations, why does a supposedly “neutral” antagonist such as flumazenil, which occasionally displays partial agonist effects, have these clear inverse agonist properties similar to the effects of beta-CCM? The question remains unanswered. One hypothesis is that flumazenil may inhibit the action of endogenous benzodiazepine-like brain compounds[8].

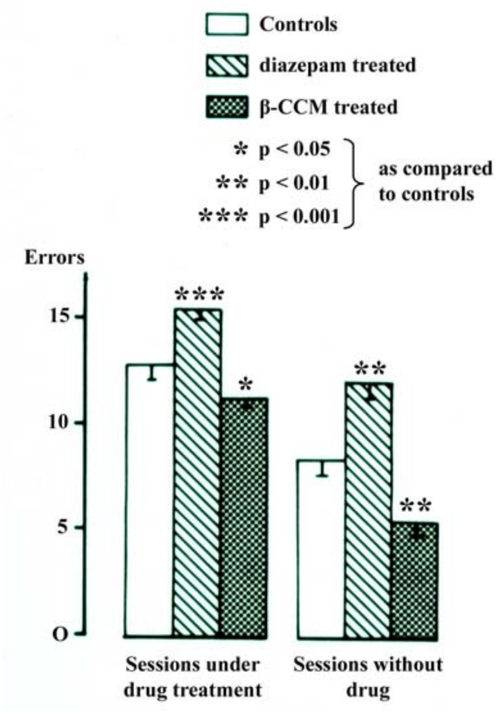

**FIGURE 7.** Results obtained in the T-maze after administration of diazepam and beta-CCM**.** For the first three sessions (30 trials), one group of mice received diazepam (2.5 mg/kg, i.p.) and one group received beta-CCM (0.3 mg/kg, i.p.); the three last sessions were performed without any drugs being administered. The results are presented for both groups and both situations (with and without drugs), and are compared to nontreated controls; they showed an increase in the number of errors by diazepam-treated animals and a decrease in the number of errors by beta-CCM–treated animals, suggesting that diazepam has an impairing action on learning and that beta-CCM has an enhancing action on learning. (Adapted from Raffalli-Sébille et al.[12].)

After obtaining these animal data with beta-CCM, attempts were made to improve memory processing in humans by using other compounds acting on the benzodiazepine site[82,83]. These compounds included benzodiazepine receptor inverse agonists, S-8510 and 2-(3-isoxazolyl)-3,6,7,9-tetrahydroimidazo[4,5-d]pyrano+ ++[4,3-b] pyridine monophosphate monohydrate[84], and a novel triazole compound, MDL 26,479[85,86], which had been suggested as treatment for memory deficits, but further research is needed to confirm the initial findings. It has been noted that antagonists, such as flumazenil, can enhance learning in animals and another antagonist, ZK-93426, appears to have produced similar effects on human volunteers[87,88].

The terms “amnesic” and “promnesic” are commonly used when referring to the clinical effects of benzodiazepines in humans[89,90]. Benzodiazepines and beta-CCM appear to act specifically on the acquisition (learning) phase of memory processing. Behavioral effects can be observed in subsequent trials testing retention session, but only when the compound is administered before (or sometimes immediately after) the first acquisition (learning) session. However, no effect has been observed when the compound is administered once only, immediately before the retention session (see Fig. 5). References to “amnesic” or “promnesic” actions, therefore, may be misleading and the terms “acquisition impairment” and “acquisition enhancement” would be more appropriate[91,92]. Even the classical expression “memory consolidation process”[93] is not entirely correct as acquisition is only the first part of consolidation.

The data described in the first part of this review led us to an interesting idea, i.e., that there should be a physiological link between beta-CCM–induced convulsions, beta-CCM–induced anxious behavior, and beta-CCM–induced learning processes, as beta-CCM, a widely used beta-carboline, is convulsant at high doses, anxiogenic at moderate doses, and learning enhancing at very low doses. A link could be postulated, therefore, between pharmacologically induced learning and mild anxiety[94]. A higher anxiety level would lead to pathological states, such as seizures[95].

The discussion here will not detail the role of GABA-A receptor subunits in these different effects, as excellent reviews have focused on this fast-changing field[96,97,98]. Evidence has been presented showing a relatively specific role for α3 and δ subunits in the control of epileptic seizures[83,84], of α2 and α3 subunits in the control of anxiety[99,100], with γ2 subunits needed for binding at the benzodiazepine site[101,102], and for α5 subunits in the control of learning processes[103,104,105,106,107]. It should be noted, however[108], that while this analytical knowledge is obviously important for the basic understanding of the different processes discussed here, it should not dominate to the point where a broader view of behavior is overlooked. Behavior patterns, e.g., in anxiety and learning, can be related to specific subunits, but there may also be a physiological link. In the case of anxiety, and in the light of data presented above, the fact that anxiety is linked to both seizures and learning processes may be relevant. Behavior is not only an accumulation of separate phenomena, but is also an integrated collection of patterns; a description of the overall functioning of the brain (and body) could be done using the term "mosaic" (commonly used in other fields of biology) to designate simultaneous autonomy and integration of component parts[109]. Current knowledge of the role of GABAergic neurotransmission in anxiety tallies with such a biological interpretation.

## TWO MOUSE STRAINS SELECTED FOR THEIR SENSITIVITY TO A BETA-CARBOLINE

### Strain Selection

Two strains were selected for their response to a single dose of beta-CCM, one for sensitivity (BS) and one for resistance (BR)[110]. For the purpose of simplicity, instead of selecting the mouse strains for other more complex behavioral traits, the strains were selected on the basis of the convulsive effect of a single dose of 4 mg/kg, i.p., of beta-CCM[111]. After administering beta-CCM, a seizure usually occurs within 2–5 min and is often preceded by clonic movements of the limbs and/or vibrissae[32]. The seizure has a very recognizable behavior pattern, with tonic-clonic movements throughout the body, until the animal falls on its side[32]. The entire episode lasts a few seconds. Compared to more complex behavioral phenomena, such as anxiogenic responses or a learning paradigm usually involving observation ranging from 5 min to several hours, seizuring is a practical way of selecting for their sensitivity/resistance to beta-CCM.

Selection[111] was made from a heterogeneous, but genetically controlled, starting pool, crossing four strains known to be resistant to beta-CCM (C57BL/6J, C57BL/10J, XLII, NZB/B1NJ) and four strains known to be sensitive (BALB/cBy, CBA/H, C3H/HeJ, DBA/2J) to beta-CCM. All the strains were bred from genitors originally provided by Jackson Laboratory (Bar Harbor, ME), except for strain XLII, provided by CSEAL (Orleans La Source, France). Seven of the strains were chosen on the basis of data previously obtained in our research group[112] when testing a slightly higher dose of beta-CCM (5 mg/kg, i.p.). The C57BL/10J strain was chosen on the basis of unpublished findings from our laboratory.

The dose of 4 mg/kg was chosen as the most discriminative, as seen with preliminary observations; a ceiling effect had been reported at 5 mg/kg for sensitive strains (100% seizures)[112]. Breeding was as follows:

- Four crosses were performed (C57BL/6J × BALB/cBy; C3H/HeJ × C57BL/10J; XLII × DBA/2J; CBA/H × NZB/B1NJ).

- Three months later, further crosses were performed with three females from the C57BL/6J × BALB/cBy F1 cross-mating with one male from the C3H/HeJ × C57BL/10J F1 cross, plus three CBA/H × NZB/B1NJ F1 females mating with one XLII × DBA/2J F1 male.
- Another three months later (+6 months), 25 breeding pairs from the last generation were intercrossed and the offspring of the next generation formed the starting population (G0).

Each mouse in the G0 starting population was individually tested for beta-CCM sensitivity, using a dose of 4 mg/kg, i.p.[111]. The response was assessed over a 6-min observation period. The selection was made for beta-CCM sensitivity/resistance and three groups of 10 breeding pairs were kept:

- One group of 10 males and 10 females not convulsing at all within the 6-min time limit; these were classified as the beta-CCM resistant group (BR).
- One group of 10 males and 10 females that recorded the longest seizure latencies: the beta-CCM moderate response group (BM).
- One group of 10 males and 10 females with the shortest seizure latencies: the beta-CCM sensitive group (BS).

Pairs were caged to mate and breed the first generation offspring (G1) selected for the present study. The breeding continued, producing a new generation every 3 months: G2, G3, G4, and so on. Pairs were selected to avoid inbreeding. Fig. 8 summarizes the findings. BS-BR differences are clear and reach significance from G2 on; the chi square test on the percentage of convulsions shows $p = 0.05$ at G2 and $p < 0.001$ from G3 to G6[111]. From generation G3 on, very few animals qualified as BM, and with insufficient numbers to continue breeding, the BM group was discarded and the study continued with the BS and BR groups.

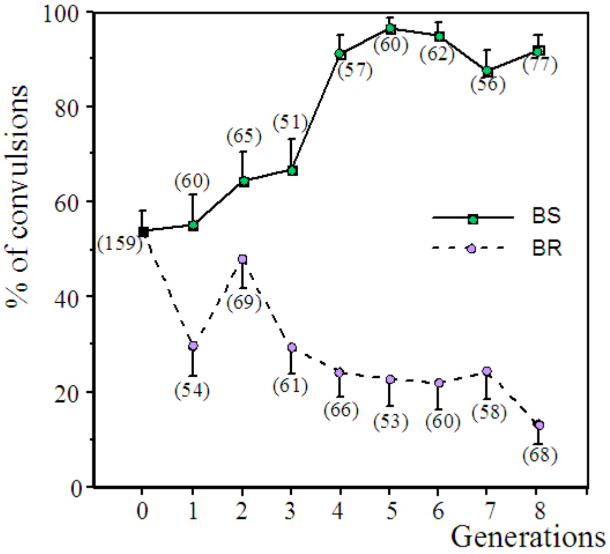

**FIGURE 8.** Selection of two mouse strains sensitive (BS) or resistant (BR) to a convulsive dose of beta-CCM. Following Mendelian principles (see text), two mouse strains were selected for their response to a single convulsive dose of beta-CCM (4 mg/kg, i.p.). The percentage of subjects in each generation is given in parentheses. The percentage of convulsing mice increased quickly in sensitive mice (BS) and the opposite was observed in resistant mice (BR). (Adapted from Chapouthier et al.[111].)

Different studies were conducted as soon as each generation became available:

- G6 — Binding studies

- G7 — Aggression studies
- G9 and G10 — Effects of other GABA receptor ligands
- G11 to G14 — Anxiety studies
- G14 — Effects of antidepressants
- G 17 and G18 — Effects on learning

Regular tests were conducted to check the persistent expression of sensitivity/resistance to the convulsive action of beta-CCM, testing a small sample with a dose of 4 mg/kg. Both males and females were used, except when targeting specific traits, e.g., aggressive behavior in males as an inclusion criterion for aggression studies and an exclusion criterion for long-term investigations. These mouse strains were selected for their sensitivity/resistance to beta-CCM–induced tonic-clonic convulsions, and the two strains bred in Orleans from BR and BS mice (respectively, BR/Orl and BS/Orl[113]) have proven to be an interesting model for absence-epilepsy; the spontaneous spike-wave discharges in the EEG recordings, characteristic of absence-epilepsy in humans, were observed in BS/Orl mice, but not in BR/Orl mice[113].

## Binding Data

Binding studies on BR and BS brains[111] were done with labeled flumazenil (a receptor antagonist binding to the benzodiazepine receptor and usually not affecting behavior). Assays were scheduled at set times after the injection and the best results were observed after 1 min had elapsed, the interval then chosen for the Scatchard analysis. No sex-related difference was observed. Controls were given a saline injection and no interstrain difference was seen for Bmax and Kd values, but clear differences were seen between BR and BS mice treated with beta-CCM: Bmax went down in both strains treated with beta-CCM, and more so in BR mice (Fig. 2). Kd values went down in treated BS mice and went up in treated BR mice. The drop in Bmax in BR mice tallied with similar observations of resistant strains: C57BL/6J[114] and TaTfm[115]. One hypothesis is that resistance to seizures may be triggered by or linked to low Bmax values. The sharp drop in Bmax may express a change in the number of receptors caused by the administration of beta-CCM, making the mice resistant to the convulsant agent. It is more difficult to develop an hypothesis for the Kd changes; this was also the case in previous studies of C57BL/6J and TaTfm mice.

## Action of Other GABA Receptor Ligands

Beta-CCM sensitivity-based selection offers scope for investigating sensitivity to other agents affecting the GABA-A receptor complex and possible coselection, e.g., sensitivity to a benzodiazepine (diazepam) and convulsant agents acting via the chloride channel (pentylenetetrazol, picrotoxin)[116]. Standard elevated plus-maze trials have been used to study sensitivity to diazepam and anxiolytic/anxiogenic effects[117], and states of vigilance have been monitored to observe its sedative effect. No sex-related difference was seen in the elevated plus-maze. Anxiolytic doses (1.5 and 2 mg/kg, i.p.) had no behavioral effect on BR mice, but produced clear behavioral changes in BS mice, with more entries and more time spent in the open arms, showing that BS mice were more sensitive to the anxiolytic effect of benzodiazepine/diazepam.

Sedation/vigilance was studied, testing males only to eliminate the effect of female cycles[118,119]. Three parameters were recorded: wakefulness, slow-wave sleep, and paradoxical sleep, testing mice after i.p. administration of diazepam, using an anxiolytic/mildly sedative dose (2 mg/kg) and a patently sedative dose (4 mg/kg). Saline-injected controls had the same wake/sleep patterns and it was concluded that there was no genetic impact on vigilance. In treated mice, no interstrain differences were observed in the 4 h after diazepam administration. Paradoxical sleep remained unchanged, perhaps because the dose of diazepam was not high enough to trigger a change. Treated BR mice showed no changes, but the period of wakefulness was much shorter and therefore slow-wave sleep lasted much longer in BS mice in the 4–8 h after diazepam injection. The delayed action may have been caused by the diazepam metabolites and not directly by the diazepam[120,121]. The conclusion drawn was that BS mice were sensitive to the sedative effect of diazepam as measured and that this was not the case for BR mice.

The sensitivity of the same two strains to the GABA convulsant receptor ligands, pentylenetetrazol and picrotoxin, was also tested and a strain-related difference was observed: 30 and 45 mg/kg, i.p., of pentylenetetrazol induced seizures in 70–80% of BS and only 0–10% of BR; and 4 and 5 mg/kg of picrotoxin triggered seizures in 90–100% of BS and only 0–30% of BR; all differences were highly significant.

While few studies have investigated this, it is interesting to note that another classical convulsive agent, kainic acid, not acting on the GABA receptor, has completely different effects on the two strains[122]. These observations support the hypothesis of breeding sensitivity (BS) and resistance (BR) to a number of GABA-A receptor complex ligands through genetic selection[116]. Any interstrain studies of animals with contrasting genetic traits must recognize that certain differences cannot be attributed to breeding and selection, but could arise from a random genetic drift. Henderson[123] set a “safe margin” to factor out genetic drift, the cut-off point being above 3 SDs, and this was applied to the present study.

## Effects on Anxiety

The two strains, BS and BR, have been studied using standard tests for anxious behavior displayed by mice: the staircase[124], light-dark discrimination[49], the elevated plus-maze[125,126], and the open field[127]. Other observations include locomotor activity, the hot-plate test to assess sensitivity to pain[128], and the hole-board test to measure exploratory behavior[129]. Male mice only were tested.

Strain-related differences were reported, but not for all tests. No significant difference was observed in the open-field trials; in the elevated plus-maze, BR mice spent more time than BS mice in the closed arms, and this was interpreted as more “anxious” behavior by BR mice. The light-dark discrimination test showed similar differences with longer latencies for BR mice leaving the dark compartment for the first time[130] and therefore less time in the lit box (Fig. 8), and fewer transitions between compartments, compared to BS mice, i.e., all behavioral responses considered to be “anxious”[49]. In the staircase test, the BR mice climbed the same number of steps, but recorded more rearings; once again, these behavioral phenomena are considered to be “anxious”[124].

Observations of locomotor activity showed BR mice had less horizontal locomotor activity than BS mice, again interpreted as “anxious” behavior; and for the hole-board test, BR mice did less head dipping when exploring holes, a sign of more “anxious” behavior. For the hot-plate test, no significant difference was observed for rearing or jumping, even though the latency time for licking forepaws was longer for BR mice, and the hypothesis of hyperalgesia in BR mice was ruled out. Most data on these behavioral parameters tally with the hypothesis of greater anxiety in BR mice compared to BS mice. Exceptions, where no difference is observed, e.g., the open-field, may arise in a novel environment when the instinctive response, which is to explore, could conceal any display of anxiety related to the situational context[110].

Interestingly, a recent selection of rats sensitive to and resistant to another beta-carboline, DMCM[131], led to results that were the opposite of our findings for two anxiety paradigms. In the elevated plus-maze and in an open-field situation, the authors showed that the most sensitive rats most likely to convulse were also the most anxious. This result supports the putative link between convulsive action and anxiety processes in rodents, and also suggests that the operational direction of the link may depend on the population or species involved. This point will be discussed below.

## Aggression and Antidepressants

The two very different fields of aggression and antidepressants have sometimes been extended to study anxiety. In most cases, anxiety correlates positively with aggressive behavior in male mice[132] and in humans, depression is often associated with anxiety in an anxious-depressive syndrome. Two short studies have, therefore, been included here and are presented after the study of anxiety.

The resident-intruder test is a recognized tool that assesses the aggressiveness of a (male) mouse in its home (cage) environment when confronted with a nonaggressive (A/J/Orl) intruder. The speed of the first attack (latency) is the parameter that measures aggressive behavior. Two experiments were conducted under similar conditions[133] and reported similar results. BR and BS mice were tested on two consecutive days; no interstrain difference was observed on the first day, but the behavior of BS mice was systematically more aggressive on the second day. An explanation of the effect occurring only on the second day may be that BS mice are more efficient in learning tasks than BR mice (see below) and therefore performed better on the second trial, but the findings of these experiments were unexpected. Given that aggressive behavior is often associated with anxiety, and that BR mice have been found to be more anxious in a number of situations, a higher level of aggressiveness would have been expected in BR mice. However, the link between aggressive behavior and anxiety is only statistical, and as is the case here, there are exceptions to the rule.

Two animal models classically chosen for studies of antidepressant compounds are the forced-swimming test[134] and the tail suspension test[135,136]. They have been used with both BR and BS strains to assess the effect of antidepressants[137]. In the forced-swimming test, the more "resigned" or "depressed" mice swim less and are therefore immobile for longer periods, a behavior pattern that can be modified by administering antidepressants. The tail-suspension test produces the same type of immobility in "resigned" or "depressed" animals that register fewer and less powerful movements. In both tests[137], BS mice recorded more immobility time than BR mice. Trials of the antidepressant imipramine were conducted using the tail-suspension test and by treating the mice with two successive doses of 30 mg/kg, i.p., 5 h and 30 min before the trial. There was a significant reduction in immobility time in imipramine-treated BS mice, but no effect was observed in BR mice. The general observation is that BR mice display fewer signs of being depressed or resigned than the BS strain, but due caution must be exercised when extrapolating to humans, as no animal model can perfectly mimic human pathologies[126]. Here again, the link with anxiety was the opposite of what might have been expected: anxious BR mice appeared to more resistant to depressive-like situations than nonanxious BS mice[110].

## Effects on Learning

Evidence has also been presented of differences in the learning abilities of the two strains[138]. Three different learning tasks were used: spatial delayed discrimination on the four-hole board, a learned choice between a lit and a dark arm in a T-maze, and place learning in an eight-arm radial maze. In the four-hole board task, four holes on the floor opened onto a food container. Photocells placed in each hole recorded the number of head dips. Subjects were kept at 80% of their normal weight. During the acquisition phase, each subject was free to explore the hole board for 6 min. During the acquisition phase, food pellets were available, but only from one (rewarded) hole; the hole changed from one subject to another. The postacquisition test phase was carried out 5 min, 4 h, or 24 h later, using independent groups of BS and BR mice. Retention was assessed by recording moments over a 3-min period and with no pellet available, counting the number of head dips per hole and the exploration time for each hole.

In the light-dark choice situation, the learning apparatus was a T-maze, with a start arm and two other arms to choose from. The floor of each of the three arms and of the connecting hub could be electrified separately. The two choice arms were lit with a 25-W electric bulb positioned directly above the ceiling. Training involved six consecutive daily sessions of 10 trials each. The animals were trained to choose the dark arm. For each trial, an error was counted only when an animal entered the lit arm; a positive response was counted when an animal had all four legs in the dark arm. Light and darkness for the left and right arms of the maze were distributed in a Gellerman random sequence. In a learning task in an eight-arm radial maze, the apparatus was placed directly on the floor, positioned in one direction only, in a room featuring several objects that could be used as cues for spatial orientation. The end of each arm was closed by a perforated partition behind which food pellets were hidden. The pellets could be reached at the end of three reinforced arms and could not be reached in the other five arms. After an habituation period, the animals that were kept at 80% of their normal weight were trained for 6 days. The mouse could only reach the food reward in three of the eight arms: two adjacent arms and another separated by a nonbaited arm (e.g., one, two, and four). Each animal had to learn a different combination of three baited arms and was tested over 6 days, with six trials per day, separated by a 1-min interval. The trial ended when the animal had reached the available pellets in all three arms. With six trials, for each animal and three baited arms, the perfect minimum score was 18 arms visited.

In all three tasks, BS mice had consistently better performance levels than BR mice (Fig. 9). In the spatial delayed discrimination task on the four-hole board, BR and BS mice had a similarly high percentage of recognition after a 5-min interval; but at the 4- and 24-h intervals, BR mice appeared to be operating at random, and BS mice registered better performed for the same time intervals; the difference was significant. It was concluded that BR mice had a faster time-dependent rate of forgetting compared to BS mice. In the other two experiments, the light-dark choice (Fig. 10) and the eight-arm radial maze, BS mice consistently made fewer errors than BR mice.

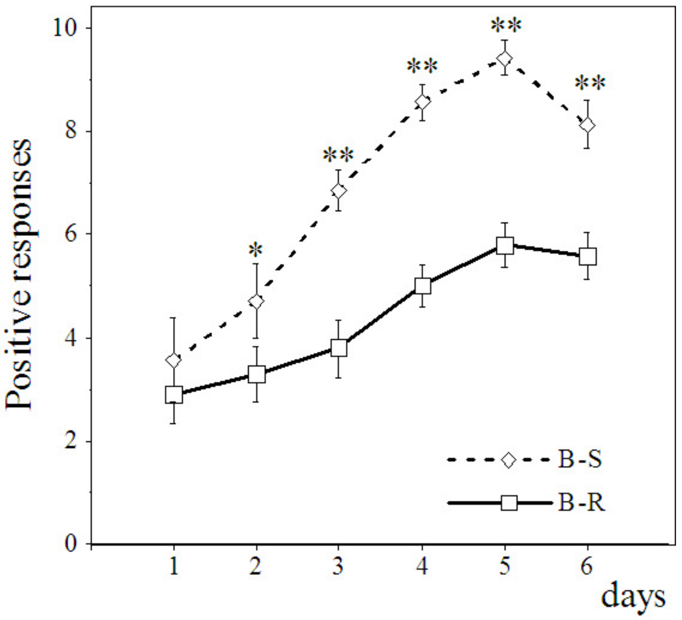

**FIGURE 9.** Learning differences in BS and BR mice**.** In the same learning situation as Fig. 6 (but with no drugs administered), BS mice learned substantially faster than BR mice. (Adapted from Venault et al.[138].)

All these data[138] tend to confirm that there is a relationship between anxious processes and learning skills.

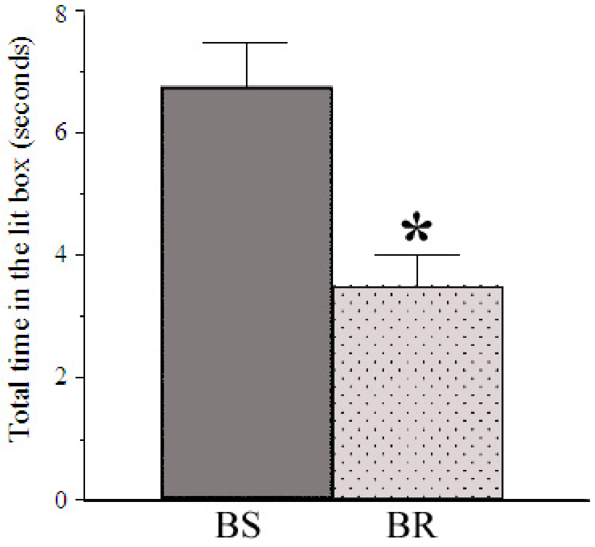

**FIGURE 10.** Reactions of BS and BR mice in an anxious situation**.** Behavior was observed for 5 min in an experimental setup comprised of two identical boxes, one dark and one lit. Anxious mice spent more time in the dark. Time spent in the lit box was interpreted, therefore, as an index of "nonanxious" behavior. The total time spent in the lit box was greater for BS mice, which led to the deduction that BR mice were more anxious. (Adapted from Suaudeau et al.[130].)

## CONCLUSION: A PUTATIVE LINK BETWEEN CONVULSIONS, ANXIETY, AND LEARNING PROCESSES

The first part of our review, emphasizing the direct effect on behavior of inverse agonists, beta-carbolines, and equivalent compounds, led to the conclusion that there might be some form of physiological relationship between convulsions, anxiety, and learning processes operating via the ubiquitous GABA-A receptor complex. Beta-CCM, for example, one of the most widely used beta-carbolines, when administered to rodents is convulsant at high doses (10 mg/kg, i.p.), anxiogenic at moderate doses (1 mg/kg, i.p.) and, in certain situations, can enhance learning at very low doses (0.3 mg/kg, i.p.). A link could thus be postulated between learning and mild anxiety[94], suggesting that the "normal" state of the brain for optimal learning would be a state of mild anxiety, although when anxiety levels are too high, there would be pathological states, e.g. seizuring[95]. This idea can be supported by the observation that the same brain area, the limbic system, has been shown to be involved in seizuring[139], anxiety[140], and memory processing[141]. However, these three processes have been associated with different type of GABA receptors subunits (as stated above). Several subunits with different actions are present in the same GABA complex and we believe this to be of evolutionary significance. The useful discovery of selective ligands of the GABA-A receptor subunits does not preclude the important role that is played by broadly and biologically active compounds targeting several subunits at the same time[108].

The behavioral study of the two mouse strains, one selected for sensitivity to beta-CCM (BS) and one for resistance (BR) to beta-CCM, may appear to confirm this hypothesis, with differences observed in anxiety and learning processes between BR and BS mice, but closer analysis of the data shows that it is difficult to distinguish clear effects[110].

First, when assessing anxiety responses in BR and BS mice, the reverse (i.e., BS mice being more anxious than BR) could have been expected as BS mice, being more sensitive to the convulsive action of beta-CCM, might also have been expected to be more anxious, and this was the finding of Conto et al. studying rats[131]. However, our study of genetically selected mice observed the opposite, suggesting that there may be a form of antagonism between sensitivity to beta-CCM and sensitivity to anxiety-producing situations. The strain the most sensitive to the convulsive and hypothetically anxiogenic effects of artificially administered beta-CCM (BS) should, however, have greater resistance to anxiety "occurring naturally”. This appears to be confirmed by the fact that BS, which is more sensitive to beta-CCM, is also more sensitive to other pharmacological agents, both anxiogenic (e.g., pentylentetrazol) and anxiolytic (e.g., diazepam), and even to antidepressants (e.g., imipramine). Further work is obviously needed to gain a better understanding of the antagonism between sensitivity to drugs and sensitivity to “normal” situations of stress, where BR and BS have opposite responses. A biochemical hypothesis has been made for the specific action of beta-CCM[95]; the binding study suggests that the reduction in the number of seizures in the resistant strain, BR, might be linked to lower Bmax levels, which, in turn, may show an adaptation with the number of benzodiazepine receptors that may help the mice to build up resistance to beta-CCM. Resistance to anxiety in “normal”, i.e., nonpharmacologically induced, situations, seen to be greater in BS mice, would therefore depend on other, different mechanisms yet to be identified. Rinaldi et al.[142] investigated a putative correlation between susceptibility to beta-CCM–induced seizures and spontaneous anxiety, conducting trials on inbred mouse strains tested in the elevated plus-maze, but failed to find any correlation between the experimental form of epilepsy and the experimental form of spontaneous anxiety. Rinaldi et al.’s study again highlights the complex and unexplained relationship between seizuring and anxiety. It might be assumed that different kinds of anxiety exist and that the processes underlying pharmacologically induced anxiety involve, to a certain extent, mechanisms that produce effects opposite to those of the mechanisms underlying spontaneous anxiety in “normal” situations. The GABA-A receptor complex is not the only mechanism that plays a key role in anxiety processes. It has been established that BR mice developed resistance to the convulsive actions of beta-CCM via GABAergic pathways, but BS mice may have developed resistance to spontaneous anxiety via quite different biochemical pathways[95]. It is possible that other biochemical processes operating in the brain and/or other transmitter systems are involved, and research needs to be conducted into their relationship with traditional GABAergic processes and anxiety. Pharmacological and behavioral investigations of these two strains are promising fields for future research.

Results on aggression and learning processes are easier to interpret. As mentioned above, several exceptions can be found to the classical correlation between anxiety and aggression. The fact that the less-anxious BS mice perform better in memory tasks is not surprising, as many learning tasks involve anxiogenic components. However, in other situations involving pharmacologically induced anxiety (not studied with BR and BS mice), BR mice would be expected to learn better. Once again, further investigations are needed to define which kind of anxiety (pharmacologically induced vs. spontaneously occurring) is likely to improve or impair learning in specific situations.

The study of the properties of inverse agonist beta-carbolines and the behavior of the two mouse strains selected for sensitivity/resistance to beta-CCM can lead to physiological hypotheses: there may be an organic link of some kind between seizures and certain types of anxiety and memory processes, operating through the GABA-A receptor complex. However, the fact that BR mice are more anxious than BS mice suggests that anxiety processes are more complex and that mechanisms other than GABA-A processes are probably involved, at least in BR and BS mice. It may be that for mice, only pharmacologically induced anxiety and learning situations affected by such anxiety are organically linked to seizuring.

## ACKNOWLEDGMENTS

The authors wish to thank Ms. Shan Benson for her help in improving the English text.
